# Women’s Healthcare Services since the COVID-19 Pandemic Outbreak in Poland

**DOI:** 10.3390/ijerph19010180

**Published:** 2021-12-24

**Authors:** Katarzyna Wszołek, Dominik Pruski, Katarzyna Tomczyk, Małgorzata Kampioni, Karolina Chmaj-Wierzchowska, Marcin Przybylski, Maciej Wilczak

**Affiliations:** 1 Department of Maternal and Child Health, Poznan University of Medical Sciences, 60-535 Poznan, Poland; katarzyna.zielin@gmail.com (K.T.); mkampioni@ump.edu.pl (M.K.); karolina.chmaj-wierzchowska@ump.edu.pl (K.C.-W.); mwil@gpsk.am.poznan.pl (M.W.); 2 Greater Poland Specialist Centre, 60-479 Poznan, Poland; dominik.pruski@icloud.com (D.P.); przybylski@lutycka.pl (M.P.)

**Keywords:** women’s health, COVID-19 pandemic, prenatal care, prevention and control

## Abstract

The COVID-19 pandemic had a direct impact on the extent of guaranteed healthcare services. Many gynecologists’, obstetricians’, and midwives’ offices were closed, laboratories suspended their activities, the collection of necessary tests was delayed, and women had to wait much longer for test results than they had to previously. General women’s healthcare prophylactic programs were suspended or delayed. In 2020, screening financed by public funds covered less than one-seventh of the female population in Poland. As medical teams, professionals, clinicians, and scientists, we have been facing a challenge to help, protect, and care for one of the most vulnerable population groups, pregnant women. A significant part of that challenge has been in preventing the spread of severe COVID-19, along with other preventable diseases, among women who are pregnant, who are in labor, or who have recently given birth.

## 1. Introduction

The first public restrictions in Poland started on 13 March 2020 when the Polish Government announced the Risk of Pandemic Status (Journal of Laws of the Republic of Poland, No 2019.1239) [1]. On 20 March 2020, the Polish Government announced the Pandemic Status (the Ministry of Health Regulation, 20 March 2020: Journal of Laws of the Republic of Poland, No 491) [2]. Gynecological and obstetrics hospitals, as well as other medical centers, were obligated to modify their procedures [3]. Detailed modes of conduct with patients were described according to recommendations by the Polish Association of Gynecologists and Obstetricians, the chairperson of the Polish Association of Neonatologists and the chairperson of the Polish Association of Perinatal Medicine [4]. Due to the pandemic, cesarean sections were advised at the beginning of the COVID-19 outbreak [5]. During childbirth, women in labor were encouraged to use a face mask to cover their nose and mouth [4]. Family-assisted childbirths were suspended in Poland on 13 March 2020. Parents’ access to their hospitalized children in neonatal wards was also suspended.

Polish recommendations regarding perinatal care were precisely described in the Regulation of the Minister Of Health, 16 August 2018, on the organizational standard of perinatal care, and have been in force since 1 January 2019 [6]. The regulations describe not only the scope of preventive services and activities in the field of health promotion, but also the diagnostic tests and medical consultations performed in pregnant women along with the periods of their performance. Preventive services provided by a doctor or a midwife, health-promoting activities, diagnostic tests, and medical consultations (with comments regarding the pandemic state) were assigned to respective pregnancy weeks [6] unfortunately the COVID-19 pandemic has had a direct impact on the extent of these guaranteed healthcare services. Many gynecologists’, obstetricians’, and midwives’ offices were closed, laboratories suspended their activities, the collection of necessary tests was delayed, and women had to wait much longer for test results than they had to previously.

## 2. The Aim of the Study

The aim of our study was to highlight the concerning trend of the cessation of prophylactic programs intended for women (especially those at high risk of cancer development) or the cancellation of medical appointments for women, especially during pregnancy and the postnatal period. We would like to emphasize the urgent need to protect and preserve the high standards for women’s healthcare that have been developing in Poland for so many years.

## 3. Materials and Methods

We used integrative review methods, as they allowed us to consider studies with varying methodologies. We also analyzed measurement data published by the National Health Fund—Regional Branches, as well as national data, to find exact numerical data.

### Search Strategy and Study Selection

We searched the Web of Science Core Collection, National Library of Medicine (PubMed), the Cochrane Database, and Embase using the following phrases “COVID-19” or “SARS-CoV-2”; “Poland” or “Polish”; “women’s health” or “recommendations”; or “prophylaxis” or “perinatal care”, from the beginning of 2020 to the current date. We screened the resulting titles and abstracts and excluded those that did not relate to women’s health (prophylaxis, recommendations, perinatal care) during the COVID-19 pandemic. Another part of our search was the analysis of the National Health Fund—Regional Branches and national data regarding breast and cervical cancer prophylactic programs, as well as the Polish government’s online legal acts system. 

## 4. Results

Initially, we searched 280 articles (searching dates: 10 October and 8 November 2021) in the Web of Science Core Collection, National Library of Medicine (PubMed), Embase, and the Cochrane Database. After removing duplicate items and articles relating to the different subject areas, only 28 articles fulfilled the relevant subject criteria. On the Polish government’s online legal acts system, we found nine legal acts relevant to this study. The National Health Fund—Regional Branches and national data regarding breast and cervical cancer prophylactic programs are available on the official website: https://www.nfz.gov.pl/dla-pacjenta/programy-profilaktyczne/dane-o-realizacji-programow/ (accessed on 8 November 2021). The remaining five articles and reference positions seemed to be essential to setting out the context of the article.

## 5. Perinatal Care

The guaranteed, mandatory preventative services, diagnostic tests, and medical consultations for pregnant women and COVID-19 impact with comments were shown in Table 1.

## 6. General Women’s Healthcare Services

General women’s prophylactic healthcare programs were also suspended or delayed. Among the female population in Poland, a crucial role is played by the prophylactic programs against breast and cervical cancer. Breast cancer is the most frequent malignancy annually (24,644 new cases in 2020, 7.4% of diagnosed women died) but cervical cancer has the highest death toll—world age-standardized mortality rates for cervical cancer ranked Poland 11th globally (approximately 5 deaths per 100,000 women/year) [11,12]. The Polish Population-Based Breast Cancer Early Detection Program is directed towards women aged 50–69 who have not had mammography within the last two years and have never been treated for breast cancer. The basic stage involves mammography of both breasts, and the extended stage involves additional tests (breast ultrasonography, targeted mammography, or fine-needle biopsy) if necessary [13,14]. The Population-Based Cervical Cancer Early Detection Program is directed towards women aged 25–59 who have had no cytology smear taken within the last two years and have never been treated for cervical cancer [15,16]. In 2020, screening financed from public funds covered less than one-seventh of the female population in Poland—through the Polish Population-Based Breast Cancer Early Detection Program, 34.94% of the population had a mammography screening; through the Population-Based Cervical Cancer Early Detection Program, only 12.65% women had a cervical smear test [17]. Another source reported that in some Polish provinces, the number of generally performed mammograms was reduced by over 90%, and cytology pap smears by over 85% in 2020 [18].

For the duration of the SARS-CoV-2 pandemic, temporary recommendations were prepared by the Polish Society of Gynecologists and Obstetricians and the Polish Society of Colposcopy and Cervical Pathophysiology on secondary prevention of cervical cancer in accordance with the guidelines of the World Health Organization. The developed recommendations allowed for the postponement of diagnostic and therapeutic procedures in patients with abnormal results of screening tests towards pre-neoplastic and neoplastic cervical conditions. Polish experts, led by Professor Robert Jach, created Colposcopy 2020 [19]. In cases where it was possible to collect material from the cervix, it was recommended to perform LBS (liquid-based screening), which allows for several diagnostic tests to be performed from one sample collection and makes possible faster qualification for colposcopy with targeted biopsy of suspected sites. In the LBS diagnostics, it is recommended to use the dependent HPV model—the primary isolated test for 14 highly oncogenic HPV types, HRHPV14, or the combined test including the HRHPV14 test and liquid-based cytology (LBC). All abnormal results require further diagnostics—either the p16/Ki67 immunocytochemical test or extended genotyping and possible colposcopy examination with targeted biopsy and curettage of the cervical canal in accordance with the colposcopy protocol used in accordance with Colposcopy 2020 [20,21]. Temporarily diagnostic and therapeutic treatment based on HSIL risk assessment is allowed as stated in the recommendations of ASCCP 2019 (the American Society for Colposcopy and Cervical Pathology). Self-sampling of the material from the cervix with the use of a dedicated brush was recommended for the primary screening of cervical cancer. Diagnostic tests registered by the FDA or validated with the VALGENT and Meijer protocols are recommended. Should it be technically impossible to perform LBS, conventional cytology may be performed. It is recommended to carry out colposcopy and biopsy within 3 months in case of HPV 16, 18, and 31; in cases of unknown results or confirmed HPV HR status during the previous 12 months; or in cases of positive p16/Ki67, ASC-H, HSIL, or AGC results. For patients diagnosed with minor screening abnormalities, it is recommended to delay the diagnosis for 6 to 12 months. If an invasive process is suspected, further steps should be taken immediately [21].

## 7. Discussion

Pregnant women, women in labor, and new mothers constitute a group that has been severely affected by the pandemic restrictions, particularly regarding difficult access to specialists, delay of planned and routine screening tests and preventive programs, lack of solutions for women in quarantine or isolation in the beginning of pandemic state, limitation of the possibility of a relative’s participation during consultations and childbirth, separation of mothers and newborns, and no possibility of staying, even with a severely sick or prematurely born child [22]. The current expert opinion in Poland stresses that family-assisted births are possible during the pandemic but some conditions have to be fulfilled and the safety of other patients and staff is crucial [23].

It is impossible not to mention many aspects related to the fear of natural childbirth among Polish women. The main source of anxiety is the labor pain, but women also report fear associated with presumptions of complications with the newborn [24]. Pregnant women struggling with fears and deprived of the supportive care provided by their partners during childbirth are in the high risk group of developing postpartum depression, anxiety, and post-traumatic stress disorder [25]. The pandemic outbreak strongly contributed to an additional source of fear—a novel SARS-CoV-2 virus with an unknown impact on maternal and neonatal health outcomes, especially among women living in areas strongly affected by COVID-19 [26,27]. Means of preparation for childbirth were modified—online meetings replaced the traditional antenatal classes, which were a great opportunity for face-to-face meetings with specialists and other parents [27]. Traditional meetings were also an opportunity for physical activity and practical physical training in preparation for childbirth [28]. Mothers may need additional mental health support, as Chrzan-Dętkoś et al. stated [29], and as professionals and caregivers, we feel responsible to indicate to them the proper solutions and support sources.

An interesting survey was conducted by Sienicka et al. [30], wherein 22% of a group of 984 participants (males, females, and non-binary individuals) changed their reproductive intentions. In this group, 86.6% were afraid of limited access to prenatal care and delivery services and 81% were afraid of giving birth at the hospital [30]. This survey, although based on an online questionnaire, shows that limitations in perinatal care have a real impact on the decisions in regard to family planning in Polish society.

The Polish Patient Ombudsman Statement, published online on 16 November 2020, emphasized that obstacles related to the pandemic in perinatal health and general women’s prophylactic healthcare programs cause a real threat to human health and life [31]. 

As medical teams, professionals, clinicians, and scientists, we have been facing a challenge to help, protect, and care for one of the most vulnerable population groups—pregnant women. A significant part of that challenge has been in preventing the spread of severe COVID-19, along with other preventable diseases, among women who are pregnant, who are in labor, or who have recently given birth.

According to the legal act of 27 August 2004 on Healthcare Services Financed from Public Funds, “persons under 18 years of age and who have Polish citizenship or who during pregnancy, childbirth, and postpartum period, have Polish citizenship and place of residence in the territory of the Republic of Poland, have the right to health benefits financed from public funds, regardless of whether they are or not covered by general (compulsory or voluntary) health insurance” [32]. However, during the COVID-19 pandemic, especially during its first months, many public facilities were emergency-transformed into single-profiled infectious hospitals dedicated for patients with a positive SARS-CoV-2 swab result [33]. This led to the cancellation and delay of appointments, suspension of national population-based cancer early detection programs, and upheaval of laboratory testing financed from public funds [8,10,17].

Over a period of almost two years, the medical teams specialized in diagnosing and treating specific diseases have gradually disintegrated, mainly in the field of planned surgical treatment procedures.

Jakubowski et al. [34] stated that because of the pandemic, over 73% of the pregnant women analyzed in their survey experienced difficulties in accessing medical care. They also emphasized that traditional face-to-face appointments were replaced by online or telehealth medical consultations, as also observed by Węgrzynowska et al. [33]. This effect on medical consultations at this scale is unprecedented in the Polish healthcare system—until the pandemic, online consultations were very rare and unpopular [35], despite legislation published in 2011 allowing this type of consultation [36]. 

Gynecological consultations and examinations in children and adolescents should be conducted with an accompanying person, a legal guardian, regardless of the recommendations that during the pandemic only patients should be admitted for a visit (without accompanying persons). Personal protective equipment (facial mask) is required, excluding children under 4 years of age (for whom masks are not recommended) [37].

Medical workers employed on hospital wards dedicated for patients with COVID-19 should continuously follow the recommended minimum requirements for units hospitalizing patients with SARS-CoV-2 infection [38].

Within the context of prevention, we would like to emphasize the impact of vaccination in terms of inhibiting the pandemic. On 26 April 2021, the Polish Society of Gynecologists and Obstetricians published their position regarding pregnant women receiving the vaccination against COVID-19 [39]. A similar set of recommendations was published by the Centers for Disease Control and Prevention, the American College of Obstetricians and Gynecologists [40], and The Society for Maternal–Fetal Medicine [41], relating to women currently trying to become pregnant, those who might become pregnant in the future, those who are currently pregnant, and those who are currently lactating [42].

As medical teams, professionals, clinicians, and scientists, we have been facing a challenge to help, protect, and care for one of the most vulnerable population groups—pregnant women. A significant part of that challenge has been in preventing the spread of severe COVID-19, along with other preventable diseases, among women who are either pregnant, in labor, or who have recently given birth. However, we regretfully have to state that the currently available data clearly indicate the negative intermediate and immediate impacts that the COVID-19 pandemic has had on women’s health in Poland. We are additionally concerned that the current economic situation may have further negative influences on patients’ access to the Polish healthcare system, both in the private and public sectors.

## 8. Conclusions

(1) According to current cervical cancer prophylactic rules, minor abnormalities found in the cytological screening smear allow for the temporary postponement of in-depth diagnostic steps.
(2) Recommendations regarding cervical cancer procedures during the SARS-CoV-2 pandemic are essential.
(3) There is an urgent need to devise Polish recommendations regarding prophylactic breast cancer programs during the SARS-CoV-2 pandemic. According to our literature review, there is currently a gap in this field.
(4) There is a need to strictly follow recommendations regarding the assessment of the risks of depression and depression-related symptoms in pregnant women and mothers after childbirth. Community midwives visiting mothers, as well as gynecologists and obstetrics, are responsible for this screening.
(5) Vaccinations against COVID-19 among pregnant and lactating individuals are recommended.

## Figures and Tables

**Table 1:** Mandatory preventative services, diagnostic tests, and medical consultations with comments regarding the pandemic state [6].

Preventative Services, Diagnostic Tests, and Medical Consultations During Pregnancy ^1^	Up to 10th Week of Pregnancy	11th–14th Week of Pregnancy	15th–20th Week of Pregnancy	18th–22nd Week of Pregnancy	21st–26th Week of Pregnancy	24th–26th Week of Pregnancy	27th–32nd Week of Pregnancy	33rd–37th Week of Pregnancy	38th–39th Week of Pregnancy	Immediately after 40 Weeks of Pregnancy
Physical examination	+	+	+		+		+	+	+	+
Blood pressure measurement	+	+	+		+		+	+	+	+
Mammary glands palpation	+							+		
BMI determination	+									
Pregnancy risk determination	+	+	+		+		+	+	+	+
Promotion of healthy lifestyle	+		+		+		+	+	+	
Providing information about the possibility for the genetic testing	+									
Obligatory obstetrician consultation if care provided by a midwife	+									
Gathering data concerning the lifestyle and eating habits, including the consumption of alcohol and other stimulants	+									
Blood type, Rh	+									
Red blood cells antibody screen/antibodies	+					+	+			
Complete blood count	+		+					+	+	
General urine test	+		+					+	+	
Cervical cytology test	+									
Fasting blood glucose or oral glucose tolerance test (OGTT) in women with risk factors for gestational diabetes mellites (GDM)	+									
Venereal disease research laboratory (VDRL) test	+							+		
Recommendation of dental consultation	+									
Human immunodeficiency virus (HIV) and the hepatitis C virus (HCV) tests	+							+		
Hepatitis B (HBS) antigen								+		
Toxoplasma gondii immunoglobulins test (IgG, IgM) without the presence of IgG antibodies before pregnancy	+					+				
Rubeola immunoglobulins test (IgG, IgM) if the woman has not been vaccinated	+									
Thyrotropin (TSH) level	+									
Body weight measurement		+	+		+		+	+	+	+
Assessing the risk of depression development and depression symptoms if occurred		+						+		
Ultrasound examination accordingly to the Polish Society of Gynecologists and Obstetricians (PTGiP) recommendations [7,8,9]		+ ^2^		+ ^2^			+ ^2^			+ ^2^
Commencement of the antenatal education					+ ^3^					
Fetal heart rate (FHR) assessment					+		+	+	+	+
Obligatory obstetrician consultation in case of health care provided by a midwife	+					+			+	
Fasting oral glucose tolerance test						+				
Oral glucose tolerance test (OGTT)						+				
Anti-D immunoglobulin administration							+			
Obstetric examination									+	
Pelvic dimensions assessment								+		
Fetal movement assessment									+	+
Detection of a group B Streptococcus (GBS—vaginal and rectal swab)								+		
HCV test								+		
Cardiotocography (CTG)										+
Others										+

^1.^ Many gynecological/midwifery clinics were closed for a few months, appointments were cancelled, laboratories were closed, and test results were delayed. ^2.^ Only asymptomatic patients (without any cold/flu-like symptoms) with a negative history of COVID-19 should have ultrasounds. Patients with a negative epidemiological history, not feverish but with symptoms of an infection—postpone the examination until those symptoms will have disappeared. In mild cases, setting the deadline in 7 days is sufficient [8]. Modification of routine examination depending on COVID-19 infection status/screening positive for symptoms [9]. ^3.^ Usually provided by midwives consociated in clinics. Meetings with parents were suspended for a long time, some midwives began online-led antenatal classes only [10].
